# Pediatric sarcoidosis: report of seven cases

**DOI:** 10.1186/1546-0096-9-S1-P38

**Published:** 2011-09-14

**Authors:** Yépez Ricardo, Malagon Clara, Olmos Carlos

**Affiliations:** 1 Pediatric Rheumatology fellowship program, Faculty of Medicine, Universidad el Bosque, Bogotá, Colombia

## Background

Sarcoidosis is a rare chronic granulomatous disease of unknown cause. It involves different organs and shows a variable clinical presentation according to the age of onset. High index of clinical suspicion and a positive biopsy are needed to confirm the diagnosis. Immunosuppressive treatment is required. The type of organs affected and response to treatment determine prognosis.

## Aim

To describe the clinical features and course on a cohort of Colombian children with sarcoidosis.

## Methods

Retrospective descriptive study at 4 pediatric rheumatology centers in Bogota

## Results

5/7 patients had early onset(before 5^th^ birthday) another 2 had late onset sarcoidosis. All patients had a delayed diagnosis and were treated for other diseases. Early onset sarcoidosis was more multisystemic. Arthritis and uveitis were more common. The triad of: skin, joint and eye compromise was observed. The frequency of lung involvement was similar on both groups and 1/7 had mediastinal involvement. No patients with Löefgren triad were identified. A course with flares and remissions was a cardinal feature.

**Table:** 

	Early onset	Late onset
Sex(F:M)	1:1.5	1:1
Mean age of onset	2,8	6
Skin	100%	50%
Lungs	40%	50%
Mediastinal	0%	50%
Joints	100%	50%
Eye	80%	50%
Fever	80%	0%
Mean time to diagnosis(years)	3,8	3,5

## Conclusion

Sarcoidosis shows variable clinical presentations. Early onset sarcoidosis presented more febrile and multisystemic than late onset. Joint, eye and skin were more common. Histological confirmation is needed to rule out other entities. Chronic uveitis and polyarthritis determined poor outcome. Chronic course was associated with worse prognosis.

